# Strengthening health systems in Africa: a case study of the Kenya field epidemiology training program for local frontline health workers

**DOI:** 10.1186/s40985-017-0070-7

**Published:** 2017-10-24

**Authors:** Zeinab Gura Roka, Jane Githuku, Mark Obonyo, Waqo Boru, Tura Galgalo, Samuel Amwayi, Jackson Kioko, David Njoroge, James Anthony Ransom

**Affiliations:** 1 Field Epidemiology and Laboratory Training Program, Nairobi, Kenya; 2 African Field Epidemiology Network, Nairobi, Kenya; 3 Disease Surveillance and Response Unit, Ministry of Health, Nairobi, Kenya; 4 Division of Preventive and Promotive Health, Ministry of Health, Nairobi, Kenya; 5 Human Resources Department, Ministry of Health, Nairobi, Kenya; 6 Piret Partners Consulting, 611 Pennsylvania Avenue SE, Unit 358, Washington, DC 20003-4303 USA

**Keywords:** Field epidemiology, Workforce development, Preparedness, Training, Competencies

## Abstract

The logistical and operational challenges to improve public health practice capacity across Africa are well documented. This report describes Kenya’s Field Epidemiology and Laboratory Training Program’s (KFELTP) experience in implementing frontline public health worker training to transfer knowledge and practical skills that help strengthen their abilities to detect, document, respond to, and report unusual health events.

Between May 2014 and May 2015, KFELTP hosted five training courses across the country to address practice gaps among local public health workers. Participants completed a 10-week process: two 1-week didactic courses, a 7-week field project, and a final 1-week course to present and defend the findings of their field project. The first year was a pilot period to determine whether the program could fit into the existing 2-year KFELTP model and whether this frontline-level training would have an impact on local practice. At the end of the first year, KFELTP certified 167 frontline health workers in field epidemiology and data management. This paper concludes that local, national, and international partnerships are critical for improving local public health response capacity and workforce development training in an African setting.

## Background

Frontline public health workers in local governmental public health agencies have always needed an ever-evolving set of skills and knowledge to perform their jobs effectively [1, 2]. To address this global challenge, the US Centers for Disease Control and Prevention (CDC) partnered with Ministries of Health (MoH) within Kenya to implement a 2-year training program modeled on its own Epidemic Intelligence Service process [3, 4]. Kenya began its Field Epidemiology and Laboratory Training Program (FELTP) in 2004, with those enrolling receiving a master’s degree in field epidemiology from Jomo Kenyatta University. Various observers have credited Kenya’s FELTP (KFELTP) with important achievements such as responding to deadly outbreaks of Rift Valley Fever [5], cholera [6], and improving environmental health [7].

Despite the outbreak and health event response accomplishments of KFELTP, donors believed that the process for producing epidemiologists had become too academic and less capable of providing practical, real-world opportunities for participants to implement what they learned in class. Partners and stakeholders realized that this advanced-training model was too slow to achieve specific public health goals, such as assuring one epidemiologist per every 200,000 people in every country [8].

Furthermore, appropriate training of frontline public health workers on emerging infections and health conditions, including basic statistics, data collection, and data analysis, is essential for consistent implementation of the global health security agenda. In response to these concerns, KFELTP developed and implemented variants of the advanced-level training, as described in this paper.

## Case presentation

To address the call for quicker and shorter, yet comprehensive, field epidemiology trainings, KFELTP collaborated with the School of Public Health of the University of Nairobi to implement field epidemiology training for fifth-year medical students. This pre-service training program is known as the Medical Education Partnership Initiative (MEPI) and is an elective in public health and disease surveillance training [9–11]. MEPI is a 2-month training that includes 1 week of classroom instruction and 7 weeks in the field, where participants are attached to a national program office or a county health facility. The didactic component covers introductory sessions on epidemiology, surveillance, biostatistics, public health communications, and methods of outbreak investigations.

As another mechanism for training field epidemiologists, KFELTP conducted a series of short-course trainings for district medical officers. The short courses provided training in outbreak investigations and the use of evidence-based decision-making in public health. During each short-course, participants received a project assignment and they reported their findings after 3 months.

The overall objective of these courses was to help strengthen the capacity of district medical officers to plan, implement, monitor, and evaluate public health surveillance systems for priority diseases in their local region. These short courses helped to progressively build a roster of skilled health field staff in outbreak investigation and response within the target districts. These staff could be deployed anytime to fight recurrent epidemics such as cholera.

However, these two training mechanisms were external to the advanced-level FELTP and not fully integrated into the pipeline of creating functional field epidemiologists because these were one-off trainings with minimal follow-up and support for those who completed the trainings. Moreover, these advanced trainings produce between 12 and 20 graduates every year, but this number is not sufficient to provide the critical number that is needed to have impact on the public health systems especially at sub-national levels.

### Integrated field epidemiology training: the tiered model of field epidemiology training

**Fig. 1:**
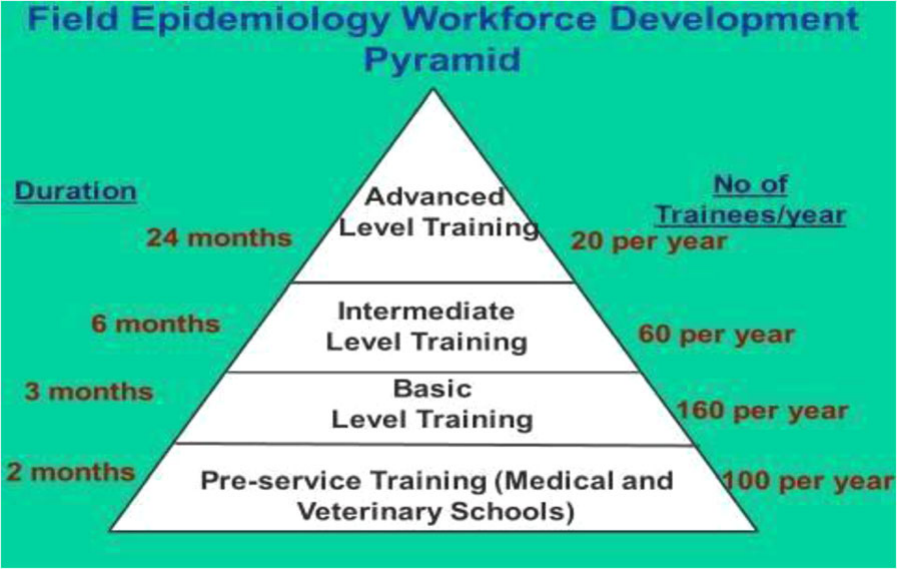
The current pyramid model of field epidemiology training and public health workforce development being implemented as part of KFELTP

**Fig. 2.**
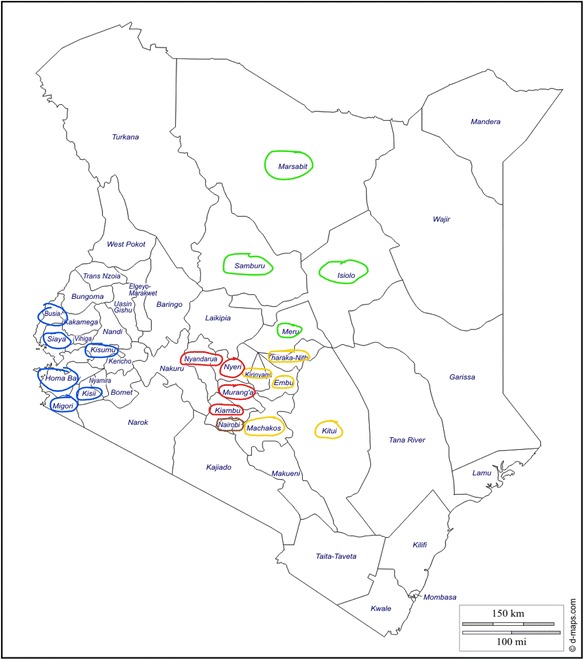
Map of Kenya’s 47 counties; counties that were clustered to form one training group are circled in the same color

After attempting to produce epidemiologists via the fragmented efforts, the KFELTP focused on a more strategic, integrated method of local capacity building to create a pathway to achieve multiple goals. In 2014, the World Bank, Defense Threat Reduction Agency (DTRA), and the CDC invested in expanding the KFELTP to include training of local frontline public health workers within their area instead of pulling them out and into the capital city, Nairobi. To coordinate and improve the preparedness of their local workforce, Kenya’s MoH adopted the tiered approach to field epidemiology training, of which frontline field epidemiology training became the second tier (Fig. 1). Political changes within the country helped fuel the MoH’s interest in this tiered approach. In 2010, Kenyan voters approved a new constitution. This new constitution stipulated decentralization from the national government to a county-specific model of governance to improve local autonomy [12]. The MoH had to configure a process to train frontline public health workers who were now employed by county health departments instead of the national MoH. The new county-based system and the clustering of counties for training are outlined in Fig. 2.

The implementation team consisted of existing FELTP faculty, with the deputy program head charged with leading the effort, and one external CDC advisor. KFELTP assumed that localization of the training would increase efficiency by consolidating resources and aligning these efforts with Kenya’s new decentralized political and governmental structure [13]. Few local health departments are likely to have the capacity needed to address the root causes of systemic problems with surveillance, disease detection, reporting, and improving the quality of health facility data [14]. For FELTP-Basic, KFELTP focused on teaching (1) introductory epidemiology and disease surveillance, (2) basic summary statistics as applied to surveillance and outbreak data, (3) basic data manipulation procedures via MS-Excel, and (4) aspects of animal-human health integration. These and other skills and topics that make up the curriculum for the basic level training are given in Table 1.

**Table 1:** Topics for didactic component of FELTP-frontline

Item	Topic	Course	Learning objectives
1	Introduction to epidemiology	1	Understand frontline epidemiology concepts: person, place, time; agent, host, environment
2	Introduction to surveillance	1	Understand frontline surveillance concepts: active vs. passive
3	Introduction to statistics	1	Understand frontline concepts of public health statistics
4	Descriptive statistics: measures of central tendency	1	Understand how to calculate the measures and interpret
5	The world of data—collection, management, analysis	1	Understand the activities associated with each aspect of training
6	Field project—data quality audit	Interim	Application of standard tool to assess the quality of data generated within their health facility or agency
7	Measures of frequency	2	Understand how to calculate rates, ratios, and proportions
8	Outbreak investigations	2	Understand the steps to undertaking an outbreak investigation
9	Monitoring and evaluation	1 and 2	Understand how to develop and implement an M&E plan within the context of public health practice
10	Communicating and presenting public health data	2	How to organize and analyze, develop visual displays, and communicate public health data
11	Field project presentations	2.5	Application of item 10 to their field project data/findings

#### Organization of Kenya’s FELTP-Basic

FELTP-Basic focuses on public health staff functions instead of public health titles, because frontline training is concerned with frontline staff who respond to health emergencies, interact with and manipulate local health data, and those responsible for reporting of surveillance data. The length of the basic training is 10 weeks, and it requires that the participants remain on their jobs. The training course is divided into two 1-week didactic sessions that are structured around short lectures, exams, case study group assignments, interactive learning exercises, and daily homework assignments. The use of case studies and learning exercises help to reinforce learning [15–17]. The mission of the case study teams is to enhance collaboration and communication. These exercises help them know how to apply newly acquired skills and knowledge back in their workplace to build and support local public health capacity to prevent, prepare for, and respond to public health events in coordination with local and regional actors and national entities [18].

#### Approach

FELTP-Basic faculty members decided to regionalize the basic trainings to (1) improve access to a broader scope of public health workers and (2) encourage buy-in and support of local county governmental structures. This regional approach was also consistent with realigned health priorities due to the devolution of decision-making to county health departments [13]. The faculty limited the number of training hubs for security and access reasons too. Many of Kenya’s frontier counties such as Marsabit, Wajir, and Garissa have been sites of ethnic violence and terrorist attacks. Therefore, frontline training cannot take place in those sites and we have to look to adjoining counties to host the training. The program hosted the trainings in the largest town among the cluster of counties.

The trainings were residency-based in that all participants and faculty lived in the same hotels where the trainings took place. Because FETP-frontline uses a cross-cadre method, faculty agreed that residential learning was a better model to facilitate learning that focuses on problem-solving and understanding points of view. Many of the participants had not participated in a training that mixed multiple public health cadres in one setting using the same curriculum for everyone.

#### Selection of counties

Group 1 was the pilot group, but subsequent groups consisted of prioritized counties that (1) did not have previous representation in the advanced-level FELTP, (2) had poor weekly surveillance reporting histories, and (3) had poor quality responses to recent disease outbreaks. Each “group” consisted of a minimum of four and a maximum of six counties, with each county contributing up to 10 participants. Because basic training is ongoing, eventually all counties would be covered within an 18-month cycle. It will take 1.5 years to cover all counties before faculty begin revisiting counties for new participants.

#### Selection of participants

The FELTP program contacted each county’s health director by e-mail, asking that they nominate at least 10 public health workers within their county to attend FELTP-Basic. Typically, faculty sent the request at least 6 weeks in advance of the proposed training date. KFELTP faculty’s goal was always to obtain a 40-person group for each training.

The FELTP-Basic selection process prioritized medical officers, veterinary officers, nursing supervisors, clinical officers, laboratory staff, and then the spectrum of public health officers (immunization managers, environmental health officers, and surveillance officers). This method of prioritization was consistent with the training and human resources development strategy issued by the MoH in 2014 [19]. KFELTP also followed these guidelines because of the sheer number of public health officers, environmental health officers, and surveillance officers populating Kenya’s public health workforce [20]. Clinicians, veterinarians, and laboratorians were prioritized because of the leadership roles they serve in their sub-counties regarding field investigations, their interaction with health facility data, monitoring and evaluation responsibilities, management of work teams and programs, and application of policy at the local level [21].

FELTP-Basic enrolled 184 participants from 27 counties between May 2014 and May 2015, and 167 (91%) of them graduated. Table 2 presents a brief profile of the year-one participants. The participants were well-educated (more than half had a university degree) with a noticeable level of professional experience (two-thirds had more than 5 years of public health work experience before the training). Furthermore, 31% were clinical staff, whereas 47% were public health officers, 16% were laboratory, and only 4% were veterinary officers. Although most participants have > 5 years of tenure, they do not have a lot of training in field epidemiology and data management. As reported by other studies, most of the public health trainings that the frontline workers participated in were vertical and program-specific trainings for HIV, TB, and malaria [22]. FETP-frontline is horizontal training that focuses on competencies outlined by the World Health Organization and its essential public health functions [23].

**Table 2:** Profile of year 1 participants (*n* = 184)

Cadre	Medical officer (10%)
	Nurse (15%)
	Clinical officer (6%)
	Public health officer (17%), environmental health officer (5%), surveillance officer (15%)
	Health records/informatics (10%)
	Pharmacist (2%)
	Veterinary officer (4%)
	Laboratory (16%)
Program affiliation	HIV/AIDS (14%)
	Laboratory (14%)
	Veterinary (4%)
	Tuberculosis (4%)
	Immunization (2%)
	Maternal and child health (7%), health records/informatics (9%)
	Other (16%)
Position	Program manager (6%)
	Department head (53%)
	Program officer (32%)
Years of public health experience	< 3 (13%)
	3–5 (20%)
	> 5 (67%)

#### Selection of course materials

Course materials were selected by FELTP faculty in conjunction with CDC-Atlanta staff. Criteria for course material selection were informed by feedback from previous participants’ (group 1 pilot) comments on the courses. Most of the existing FELTP instructional materials were developed for participants enrolling in the 2-year training course, which meant that faculty had to develop new introductory-level materials. These included pre- and post-tests, daily quizzes, case studies, and homework assignments. KFELTP modified existing lecture slides to make them appropriate for an audience of frontline county and sub-county health workers untrained in field epidemiology.

#### Selection of facilitators

Qualified instructors were crucial for the success of FELTP-Basic. KFELTP devoted considerable planning and logistical support to recruiting and supporting a wide variety of facilitators [24]. The main source of trainers and supervisors was FELTP alumni. Due to limited numbers of full-time staff within KFELTP, KFELTP-Basic leveraged a network of alumni from the 2-year training program to serve as facilitators, supervisors, and mentors within the basic training program. This process was successful because the FELTP alumni based in county health departments are typically in leadership positions [25]. The faculty leveraged these alumni for (1) teaching specific modules to the basic participants and (2) serving as supervisors for the field projects. The effectiveness of using alumni to teach and supervise was part of the “content transfer” component of the basic training. The alumni teachers understand the participants’ challenges and can help them succeed, having personally experienced and benefitted from the FETP training model. Use of alumni also helped FELTP in the continuation of the alumni teachers’ training by helping them learn to teach effectively and begin their pathway of influencing the next generation of field epidemiologists in Kenya. The faculty used daily quizzes and group case study assignments to measure the transfer of “content” to the participants. Content transfer is an important component of human resource development as it indicates if participants are acquiring new knowledge and skills and the likelihood of application of that knowledge and those skills once returned to the work site [26, 17].

### Monitoring and evaluation mechanisms

To assure that FELTP could measure improvements in surveillance and outbreak response attributable to the frontline training, FELTP instituted monitoring and evaluation structure at implementation. This helped to assure real-time monitoring, whereby the link between inputs, activities, and outputs were documented and used to improve ongoing implementation. The five parts of the formative evaluation activities are outlined below:

- Daily evaluation cards during the didactic components of the course;
- Course evaluation cards completed at the end of each 5-day didactic session;
- Course evaluation forms completed at the end of the 10-week basic training process;
- Pre- and post-test evaluations to determine if participants learned what faculty aimed to teach; and
- Supportive supervision via e-mail, text messages, and Skype.

Faculty used feedback from these sources to adjust the way the program was implemented, the materials used, and the topics covered. Based on feedback from the current group, faculty modified activities, materials, and methods for subsequent groups. For each course, faculty conducted daily evaluations that integrated into the overall evaluation effort. These daily evaluations helped to achieve sustainability by focusing on course logistics and operations that influence learning, participation, engagement, and uptake of information [27]. At the end of the week, faculty administered a course evaluation form. This level of monitoring helped KFELTP gauge right away participants’ attributes and attitudes, which are often neglected in evaluating training effectiveness. As the participants have extensive experience in participating in various types of training workshops and courses, they are expected to give valuable feedback and insight on program implementation [28–31].

### Modifications

- After group 1, faculty integrated the discussion about the field project into each day of course 1 to assure that participants knew how to use the data quality tools and the expectations of what they were to do during the 4-week field project period;
- After group 2, faculty separated the biostatistics lectures. Faculty no longer blended measures of central tendency and measures of dispersion. They are now two separate lectures with direct application to public health work;
- After group 3, faculty separated the Monitoring & Evaluation (M&E) lectures. Monitoring is covered in course 1 and evaluation in course 2; and
- Group 1 suggested that faculty expand the teaching of MS-Excel because of its ubiquitous use in county and sub-county public health agencies. After group 2, faculty integrated daily teaching in MS-Excel into the curriculum, with regionally relevant surveillance data. The second course taught MS-Excel by using data from the participants’ field projects.

### Resource mobilization

The first year of funding for FELTP-Basic was provided by CDC, DTRA, and World Bank sources. This external support allowed participants to attend the courses at no cost. The resources were sufficient to carry out all planned activities. With 184 registered participants, the direct cost per participant was $1911 for 12 days of didactic training, including a venue conducive to teaching and break-out meeting space, all meals, and coffee break snacks. The total direct expenditures at the end of the fiscal year was $366,960. Table 3 outlines the breakdown and percentage of the budget dedicated to specific spending categories for groups 1–5. Less easy to quantify, but clearly valuable, was the informal exchange of ideas, the opportunity for participants to interact with facilitators during the process as well as during meals and coffee breaks. Grants and administrative management was provided through the same non-profit organization, the African Field Epidemiology Network (AFENET), based in Kampala, Uganda, as for the advanced-level courses.

**Table 3:** Budget categories and direct expenses for year 1, KFELTP

Funding category	Expenditure	% of total
Administrative support (salaries and per diems)	$56,697	15
Field project (reimbursement of field activity costs)	$57,466	16
Conference packages (meeting space, lodging, and meals for participants and faculty)	$170,068	46
Dinner allowances for participants	$29,070	8
Transportation reimbursements for travel between home county and training location	$30,033	9
Materials preparation (printing and duplication)	$23,626	6
Totals	$366,960	100

The costs for such training is not likely to decrease with establishment of a functional framework for several reasons: (1) local health agencies lack the resources and infrastructure to develop and maintain distance-learning technology; (2) distance-learning focuses on didactics and does not fill the gap of hands-on mentoring that FETP-frontline provides to its participants; and (3) costs have to be measured in terms of preparedness of frontline health workers to respond to public health emergencies such as SARS, MERS, Ebola, and Zika as well as to health challenges emerging from environmental (climate change) and socio-economic (chronic disease) challenges in low- and middle-income countries (Table 4).

**Table 4:** Examples of additional FELTP-sponsored training activities, 2004–2016

Start date	Name	Duration	Current status	Target group	Credential
September 2004	FELTP	2 years	Ongoing	Doctors	MSc in field epidemiology awarded by Moi University
				Nurses	
				Laboratory scientists	
				Veterinarians	
				Public health officers	
September 2006	Field epidemiology short course	3 months	Ceased	District medical officers	Certificate of completion awarded by Ministry of Health
September 2011	Mepi	2 months	Ended in 2015	5th-year medical students	Certificate of completion awarded by the Ministry of Health
September 2014	Vepi	2 months	Ended in 2015	4th-year veterinary students	Certificate of completion awarded by the Ministry of Agriculture, Livestock, and Fisheries

*Mepi* medical epidemiology training program (pre-service), *Vepi* veterinary epidemiology training program (pre-service), *CDC* Centers for Disease Control and Prevention

### Lessons learned

During 2016, various programs have been used to strengthen Kenya’s response to emerging pathogens. Much of this was due to the Ebola emergency in West Africa and fear of its spread to other parts of the world [32, 33]. These were largely emerging infection in-service training programs sustained through short-term bi-lateral collaborations between the Kenya and foreign governments. FELTP-Basic has provided a regular forum for training frontline staff; staff who can deal with any public health emergency. The joint training of veterinarians, physicians, clinical officers, nurses, and other frontline public health workers is an effective means to address Kenya’s capacity-building goals. The FELTP-Basic has also created an opportunity for coordination between the human and animal health sectors and opportunities for FELTP alumni to offer service to the program that trained them as field epidemiologists.

Drawing on lessons from MEPI and the short courses, FELTP-Basic has established itself as an expected component of the FELTP constitution of training courses and has established a process that is politically unified and can integrate and align funding and knowledge management resources. FELTP-Basic has attracted frontline public health workers (more interest than spaces available) because their counties suffer from specific health conditions and/or diseases and do not have enough suitably trained health workers to address the challenges.

KFELTP’s experiences have provided an innovative, integrated approach to addressing training needs of clinicians and veterinarians by including a diverse set of public health workers in each group, thus leveraging their collective knowledge to solve public health problems and challenges. This integrated approach reflects the collaborative team-based stance needed to address many public health challenges [34].

Despite the successful implementation of the FELTP-frontline as a workforce development program, to date, the program has served only a fraction of public health workers in local governmental agencies. KFELTP has only just begun to assess the extent to which participants retain and apply information provided at the training. However, the formative evaluation conducted after each group finishes the process has consistently rated well. Future efforts will involve full implementation of the intermediate component of the tiered approach and evaluation of outcomes related to this program.

## Conclusion

FETP-frontline provides a practical solution to address public health capacity challenges in low- and middle-income countries. As the introductory level to the field epidemiology training process, the basic courses target a broad spectrum of public health practitioners to help (1) decrease the time between training and the operational capacity of those trained, (2) increase the number of public health workers who have public health credentialing beyond on-the-job training, and (3) provide a “pipeline” for better-prepared candidates who can enroll in the intermediate and advanced-level/degree-granting component of FELTP. The tiered model has allowed FELTP to invest in public health core capacities and competencies, collaborative partnerships, and critical long-term development of local human capital. The local-level training has been an efficient way for FELTP to start a process of developing national epidemiologic capacity from the ground up and help meet global requirements, such as the International Health Regulations core requirements [35].

Lessons learned in Kenya offer insights into ways to implement, maintain, and improve a basic field epidemiology training program. Our lessons can show other programs how to implement this training for public health workers in key areas of knowledge and skills improvement. The FELTP-Basic serves as a focal point for FELTP to reach out to local-level frontline workers and offer them opportunities for learning and interaction among public health officials and their key partners. Our experience suggests that political will, consistent administrative support, and the commitment of funding partners are essential for success. This is particularly important because FELTP-Basic is funded through agencies that are tasked with implementing the global health security agenda. FELTP-Basic can be a template for other MoHs interested in developing new programs to create a technologically, scientifically, and strategically sophisticated workforce for public health practice.
